# Personal and Work-Related Factors Associated with Good Care for Institutionalized Older Adults

**DOI:** 10.3390/ijerph18020820

**Published:** 2021-01-19

**Authors:** Javier López, Gema Pérez-Rojo, Cristina Noriega, Cristina Velasco

**Affiliations:** Department of Psychology, School of Medicine, Universidad San Pablo-CEU, CEU Universities, 28925 Alcorcón, Madrid, Spain; gema.perezrojo@ceu.es (G.P.-R.); cristina.noriegagarcia@ceu.es (C.N.); cristina.velascovega@ceu.es (C.V.)

**Keywords:** elder abuse, good treatment, humanization, institutions, long-term care, mistreatment, nursing homes, staff, person-centered treatment, residential aged care

## Abstract

Despite efforts to promote good care, many institutionalized older adults (IOA) experience elevated neglectful conditions and reduced person-centered care approaches. Based on the job demand–control model, this study aimed to analyze the relationship between nursing home professionals’ personal and organizational factors and good care provided to institutionalized older people. Data was collected through a self-administered survey completed by 208 nursing home staff members. Three dimensions of personal factors (i.e., personal accomplishment, depersonalization, and negative old age stereotypes) were significant predictors of good care. Depersonalization and negative old age stereotypes were negatively associated with IOA, and both good care and personal accomplishment were positively associated with good care in nursing homes. Only one work-related factor (i.e., management support) was positively associated with good care. Personal factors may play a significant role in good care. Management support offers a promising mechanism to promote good care among nursing home professionals. The findings support the need to change the focus on entirely completing care tasks to providing good care of residents in nursing homes that promotes management support, personal accomplishment, personalization and positive old age consideration, attitudes, and behaviors. Policies and interventions should be developed to address in a more humanized way.

## 1. Introduction

Residents in nursing homes often have many physical and cognitive problems, and the occurrence of dementia is quite frequent [1]. Most studies found that 50% of IOA have dementia [2]. Nursing homes can be a difficult environment for professionals because of the complex health and cognitive status of the residents. Family caregivers experience high levels of anxiety and depression [3]. In most occasions, family caregivers decide to institutionalize their older relative after a period of deep reflection and various consultations with specialists [4].

The poor pay and working conditions of care workers such as overwhelming workloads, lack of respect, and lack of support are well-documented, especially for nursing assistants [5,6,7]. These issues present challenges to provide good care in nursing homes. Kayser-Jones (1990) suggested a conceptual framework about quality of care in long-term institutions and described four essential aspects: personalization, humanization, no infantilization, and no victimization [8]. Quality of care among nursing staff and residents is a crucial issue to promote good care. Good care for IOA not only implies avoiding abuse (no victimization) but also promoting person-centered care (personalization, humanization, and no infantilization) [9]. Good care not only focuses on avoiding abuse. It also promotes good daily care practices, such as respect, humanization, and ethical values [10,11]. Consequently, good care includes avoiding elder abuse but is not only this, and good care also includes person centered care but it is more than this too.

### 1.1. Person-Centered Care

While the traditional long-term care model is focused on tasks and professional-directed (institution-centered perspective), the person-centered care perspective targets older people’s preferences and needs. [12]. It reflects the move from a biomedical approach based on clinical quality and quality of care to a biopsychological approach based on people´s quality of life and quality of care. The latter consists of a humanistic perspective that has been extensively used in gerontology settings in the last few decades.

Person-centered care affirms that the person should be the focus of care delivery and not their disease, frailty, deficits, nor their illnesses [13]. This perspective recognizes the importance of considering older adults, their family and staffs´ well-being and quality of life. However, different authors use the term person-centered care to refer to a variety of different concepts and there is no standard definition yet [12]. WE-THRIVE, an international consortium of long term care researchers, prioritized the following concepts within the person-centered care domain: relationship (among residents, professionals, and relatives), knowing the elder, paying attention to what is important for the person and providing a positive context in which the person can engage meaningfully. As can be observed, this consortium enhances the importance of setting caregiving goals that ensure residents´ quality of life. Considering this, there is an important international debate about what the construct good care refers to [14].

Person-centered care has found positive outcomes in nursing home staff, including organizational and personal factors. Some organizational conditions related with a higher person-centered care are a higher staff-to-resident ratio [14], a lower job turnover [14,15], better equipment and facilities [14,16], better organizational climate [17], and higher management support [12,14,18,19].

There are also individual factors related with a higher Person-Centered Care such as lower burnout [15,17,20,21], less work overload [15,21,22,23], higher intrinsic motivation [24], and less stereotypes towards ageing [14,20].

### 1.2. Elder Abuse

Different gerontologists have tried to develop theoretical models to explain a bad and good care approach. However, there is still no consensus. The difficulty in defining the causes of elder abuse in institutions and the factors associated with IOA´s good care has been pointed out [25].

Elder abuse is a common problem, commonly missed in the aging services network. It is often viewed as a ‘hidden issue’ or ‘inner affair’. In fact, elder abuse is an interpersonal violence less reported than other types of violence conducted in institutions. It is a violation of human rights that not only affects the victims but also the relatives and society in its totality. Research regarding elder abuse is still in its infancy [26,27].

Only a few studies deal with elder abuse in residential care since the trailblazing research of Pillemer and Moore (1989) [28]. Some reasons why older people are more vulnerable to suffer elder abuse in institutions are not being able to report the abuse because of their cognitive or physical difficulties, being worried about the negative consequences that may take place if they report the abuse (e.g., revenge) or feeling hopeless and believing that no one would help them [29,30]. Staff may be reluctant to admit their own or colleagues abuses for fears of reprisal [31] and they tended to condone abusive behaviors toward elderly residents [32]. Therefore, IOA and staff members are unable or unwilling to seek help. 

The direction of the abuse is varied: resident-to-staff, residents-to-residents, family-to-residents, and staff-to-residents. The last one is the most prevalent. However, a resident who experiences resident-to-resident abuse may become more vulnerable to suffer abuse by a staff member or vice versa [29]. Furthermore, studies from United States of America and European countries elder abuse is more likely to take place in a shared living environment, specifically for physical and financial abuse [33].

Ho et al. (2017), in the first meta-analysis on the global prevalence of community and institutional elder abuse estimated a prevalence of 10%. Nevertheless, this study mixed abuse in community-dwelling older people and IOA abuse [34]. Yon et al. (2019) analyzed only nine studies finding the physical and psychological abuse as the most prevalent types based on data provided by the nursing home staff. Although caution should be taken when self-reported elder abuse data is used, nearly 64% of nursing home staff acknowledges that they have abused IOA. This review estimates that there is a 33.4% prevalence of psychological abuse, 14.1% of physical abuse, 13.8% of financial abuse, 11.6% of neglect, and 1.9% of sexual abuse. All of these percentages are higher than those experienced by community-dwelling older adults [26]. However, Pillemer et al.(2016, p. 195) affirm that the prevalence of IOA abuse is not covered because of “the lack of research in this area; no reliable prevalence studies have been conducted of such mistreatment in nursing homes or other long-term care facilities” [33].

There are only a few studies that have analyzed the risk factors of elder abuse in nursing homes, and the research conducted to date on this topic is inconclusive [35]. More research analyzing the underlying risk factors is needed considering the different levels of the ecological framework. Literature has supported the role played by organizational and personal factors in elder abuse perpetrated by staff working in nursing homes. Some organizational conditions related with higher abuse are poorer working condition, particularly staffing shortages, time pressures, and lack of equipment [36,37,38,39,40], as well as lower management support, and a lack of guidance and support; a service isolated within the organization [38]. Regarding individual factors, burnout is a strong predictor of abuse [28,36,39,41]. Nursing home staff often rated work overload perception as a reason for abuse and neglect [39,40]. Abusers did not feel sufficiently motivated [37] and showed more negative attitudes towards residents [28,41].

### 1.3. Good Care

The WHO global strategy and action plan on ageing and health (2016–2020) stresses the need to provide a better long-term care to prevent elder abuse [42]. The European roadmap on healthy ageing (2012–2020) also includes strategies to improve the quality of services in nursing homes [43]. Furthermore, the European Commission suggested that desired good care levels include not only encouraging quality but also counteracting elderly abuse. Governments have the responsibility to protect vulnerable IOA and set the framework underpinning oversight of good care. Monitoring nursing homes quality has been growing in importance but needs further development. Good care implies, on the one hand, effectiveness and care safety, and on the other, patient-centeredness, responsiveness, and care coordination [11]. Professionals should work multidisciplinary and must be trained in good practices and the promotion of good care. Good care implies humanization, no infantilization, respect, and IOA empowerment.

Good care for IOA implies humanization. Dehumanization is a subtle form of mistreatment that violates basic human rights and it is even more devastating than depersonalization. Humanization follows when IOA are treated sensitively and amicably. Humane care recognizes the human attributes such as compassion, understanding and kindness. Humanization promotes sensitivity to IOA needs, especially to those with high dependency levels. Interactions are personal, where individuals are spoken to rather than spoken at [8]. 

Good care for IOA also implies the absence of infantilization, establishing an ‘adult–adult’ relationship instead of a ‘parent–child’ one among nursing home staff and older adults. IOA must be treated as adults, taking their life-long accomplishments into consideration. This includes such behaviors as avoiding scolding incontinent IOA, addressing IOA in respectful terms and dressing them in adult attire. Non-infantilization increases independency, role, and status. It also promotes and maintains a sense of dignity and self-worth. Because of the vulnerability of many IOA, there is a high risk of conducting paternalist practices in nursing homes. Being especially significant those practices related to infantilization [8].

Good care for IOA is related to respect [6,44]. Nursing home professionals’ practice implies respect for intrinsic dignity, worth, and uniqueness of each person. Respect enhances a person’s sense of dignity and pride in nursing homes [8,44,45].

The nursing–IOA interaction is positive and respectful. Nursing home staff culture promotes the interest in paying attention to and understanding older people’s deepest needs [46]. The staff also respected the family’s wishes [47]. Privacy and space are necessary so that IOA can have time with their relatives and bring closure to their lives. IOA and their families want and deserve respect and dignity [48]. Disrespect is linked, to a violation of human rights such as dignity, privacy, or autonomy [29].

Good care for IOA is related to empowerment [6]. The resident empowerment approach is well suited to helping IOA make self-selected changes. Empowerment is related with meaning, competence, and self-determination. Therefore, listening to residents empower them [49]. As a result, older people feel more meaningful, confident, and satisfied [45].

Drawing upon the job demand-control (JDC) model [50], which highlights the relevance of demands (stressors that are present in the work environment, i.e., work-related factors) and control (the potential of workers of regulating their tasks and behavior at work, i.e., personal factors), these being resources for understanding the differences in job impact between individuals, the present study aims to answer the following main research question: What is the relationship of nursing home good care with the position in the facility, work stressors (better organizational conditions and more management support) and personal variables? The hypotheses were the following: (1) team technicians (i.e., psychologists, physiotherapists, social workers, occupational therapists, nurses) will show better good care than nursing assistants; (2) The lower the levels of turnover and ratios, the more adequate the equipment and facilities, and the higher the management support, the more they will hold good care attitudes and behaviors; and (3) those professionals with less burnout, work overload, stereotypes towards ageing and more intrinsic motivation, will have a greater tendency to develop good care in nursing homes.

## 2. Materials and Methods

### 2.1. Sample and Data Collection 

We used a cross-sectional design study. A convenience sample of nursing home professionals participated. Before gathering data, the Institutional Review Board of CEU San Pablo University approved the study. We contacted several nursing homes to recruit participants. They were required to be working as a front-line care nursing home professional (staff directly involved in care). The inclusion criterion of being directly involved in care was selected because they have a close daily interaction with residents and are the largest group of professionals in nursing homes [7]. The survey was self-administered. However, trained interviewers (i.e., psychology postgraduate students and the authors of this study) assisted participants in case they needed help. Before completing the survey, interviewers explained the aims of the study, the types of questions and response options, data confidentiality, and their rights. All participants signed the informed consent. A total of 231 nursing home professionals participated in the study. Twenty-one participants did not meet the inclusion criteria (being a front-line care nursing home professional) and two participants did not complete the questionnaires and were excluded. The final sample included 208 nursing home staff members directly involved in care.

### 2.2. Measures

Questionnaires collected information on sociodemographic outcomes and the good care of the nursing home staff, as well as assessed perceived personal and work-related factors in their caregiving experiences. The sociodemographic information included was age, sex, marital status, highest education qualification attained, position in facility (nursing assistants versus interdisciplinary team technicians), nursing home equipment and facilities, non-consistent assignment of staff (turnover), and staff-to-resident ratio.

Good care was assessed using the good care scale in nursing homes (GCS-NH) [51]. Initially, this instrument was composed of 32 items (reverse-scored and direct scored) grouped in four dimensions: humanization (9 items; bonding, connection, tenderness and closeness), non-infantilization (10 items; consideration of older people as adults, avoiding overprotection), respect (7 items; respect and avoid stigmatization by staff), and empowerment (6 items; promotion of older people´s decision-making and choices and control over their lives). The items are scored on a five-point Likert scale (from 0 = nothing to 4 = a lot). This scale is based on the perspective of centered care by including practices in line with avoiding mistreatment and power relationships as well as providing individualized care, considering older people’s singularity. This scale is focused on protecting from disrespect (violation of human rights such as dignity, privacy, or autonomy). Internal consistency for this scale in this study was 0.714 (Cronbach’s α).

Burnout (personal variable) was assessed using the Maslach Burnout Inventory (MBI) [52]. It is a 22-item measure grouped in three subscales: emotional exhaustion (EE), depersonalization (DP), and personal accomplishment (PA). Research conducted in nursing homes and other healthcare contexts has extensively supported its validity and reliability [52,53]. Participants had to indicate the frequency they experienced 22 statements of ‘job-related’ feelings on a seven-point Likert scale ranging from 0 (‘never experienced such a feeling’) to 6 (‘experience such feelings every day’). The EE domain has nine items, the DP domain five items, and the PA domain eight items. High levels of EE and DP scores, and low levels of PA are associated with more burnout. Subscales internal consistency were as following: 0.866 for the EE scale, 0.728 for the DP scale, and 0.736 for the PA scale (Cronbach’s α). Internal consistency for this scale in this study was 0.710 (Cronbach’s α).

Professional quality of life was assessed using the PQL-35 Questionnaire [54]. It is a 35-item measure of the professional quality of life with three domains: work overload (WO), intrinsic motivation (IM), and management support (MS). This questionnaire is based on Karasek´s demand-control model formulated [50]. Professional quality of life was related to the balance between work demands and the perceived ability to carry them out. WO and IM are personal variables and MS is a work-related variable. Professional quality of life was assessed in a 10-point scale from 1 (‘none) to 10 (‘a lot’). The WO domain has 11 items, the IM domain has 9 items, and the MS domain 13 items. The final item that measures global quality of life and the item that measures ability to disconnect from work were excluded in line with previous studies [55,56]. Subscales internal consistency were as following: 0.845 for the WO scale, 0.815 for the IM scale and 0.918 for the MS scale (Cronbach’s α). Internal consistency for this scale in this study was 0.765 (Cronbach’s α).

Negative old age stereotypes (personal variable) were assessed using the Negative Stereotypes Towards Ageing Questionnaire [57]. This scale has 15 items. Response options range from 1 (’strongly disagree’) to 4 (‘strongly agree’). Higher scores show high levels of negative stereotypes towards older people. In the present study we found a global internal consistency index for this scale of 0.897 (Cronbach’s α)

### 2.3. Data Analysis 

Hierarchical multiple regression was used to assess the contribution of position in facility indicators; personal and work-related factors to IOA good care scores. Variables were entered into the regression equation in three blocks: position in facility (nursing assistants versus interdisciplinary team technicians) was entered first, followed by four work-related factors and then followed by six personal factors, using the SPSS software (version 24, IBM Corp. Armonk, NY, USA). We controlled the effects of position in facilities (nursing assistants versus interdisciplinary team technicians) by entering them in the first step of the hierarchical multiple regression analysis.

## 3. Results

### 3.1. Sample Characteristics

Participants were 208 staff members at 11 different nursing homes in Spain (about 19 professionals at each institution). As shown in Table 1, the mean age of participants was 39.28 years. Most participants were female, nursing assistants and had at least a high school degree and a little less than half were married.

Regarding organizational factors, the mean score for management support was 6.05. Overall, about 72.6% of respondents considered adequate nursing home equipment and facilities but only 24.5% considered adequate staff-to-resident ratios. In terms of non-consistent assignment of staff, almost 80% of participants experienced turnover.

Regarding individual or personal factors, the mean score for the burnout dimension was 10.78 for emotional exhaustion, 5.03 for depersonalization, and 40.52 for personal accomplishment. Applying the cut points, only the mean score of personal accomplishment implies high levels of this dimension. The mean score of 4.84 for work overload and 8.63 for intrinsic motivation, on a 1–10 scale, indicates a relatively high tendency to experience intrinsic motivation. Mean scores for negative old age stereotypes and good care indicated a medium level of experienced stereotypes and good care behaviors and attitudes.

### 3.2. Role of Organizational and Personal Factors on Good Care

The hierarchical regression results are displayed in Table 2. In step one, position in facility explained 6.1% of the variance (Adjusted R^2^) in good care. Position in facility and organizational factors in step two accounted for 20% of the variance (Adjusted R^2^); an increase of 14% from step one. In the final step, position in facility, organizational and personal factors explained 31.1% of the variance (Adjusted R2); an increase of 11% from step two.

Higher levels of management support were related to higher levels of good care (β = 0.259, *p* ≤ 0.01). Similarly, personal accomplishment was positively associated with good care (β = 0.243, *p* ≤ 0.001). Higher depersonalization was associated with lower levels of good care (β = −0.186, *p* ≤ 0.05). Additionally, higher negative old age stereotypes was significantly related to lower levels of good care (β = −0.242, *p* ≤ 0.001). 

## 4. Discussion

We aimed to examine the association between personal and work-related factors and the good care provided to residents by nursing home staff. As predicted, many personal or individual factors were related to good care levels in the expected directions. Nevertheless, only one work-related variable was positively related to good care: management support.

Good care models [8,9] were linked with the job demand–control model because they highlight the relevance of demands (work-related factors) and control (personal factors) as factors that seem responsible for good care, avoiding elder abuse and promoting person-centered care. Our results confirm the relevance of demands and control for good care.

Our findings also show that personal variables can have significant effects on good care. Previous studies have supported the negative effects of personal variables on person-centered care [15,17,20,21] and on risk of abuse [28,39]. The novelty of this study lies in the more in-depth description of the potentially harmful effects of personal variables on abuse and the potentially beneficial effects on person-centered care to an under-studied variable; good care. In this regard, this study stresses the importance of promoting personal variables and, more specifically, good care within the nursing home staff.

An interesting finding was that lower depersonalization and higher personal accomplishment predicted IOA good care, whereas emotional exhaustion did not. This result may be related to the importance of connectedness and empathy in nursing homes. Personal accomplishment is connected with empathy, attitudes, and behavior towards IOA care. Professionals reporting more personal accomplishment also showed more staff–resident interactions [58,59]. Depersonalization was negatively related to willingness to help [59].

Moreover, our results agree with a previous study in which only some burnout factors were predictive of person-centered Care [17]. Also, some good care trainings among professional caregivers in nursing homes had no effect on emotional exhaustion [19,21].

Supporting our hypothesis, a negative association between old age stereotypes and good care was observed. Lower levels of old age stereotypes were found to be associated with reporting higher levels of good care among the assessed individual factors. In line with these results, other studies have shown a use of stereotypes in disrespect or generally treated older adults [45], that may be related to a less effective IOA abuse recognition ability among nursing home professionals [35], and that involves more nursing home mistreatment [41]. Furthermore, there is evidence for a dysfunctional nursing home caregiving type, named rough handling care, in which professionals behave impatiently, ignoring and treating IOA as objects, or even threatening them. Moreover, nurses´ negative stereotypes affect negatively the delivery of IOA care [30]. Because of the important role played by professionals´ negative self-perceptions of aging explaining good care in this study, it should be considered as a key dimension. Given that negative stereotypes can reduce the potential IOA good care, more research and interventions should be developed among front-line professionals working in nursing homes. Institutions supporting continued education and care about reducing nursing staff´s negative old-age stereotypes, have the potential to impact on IOA good care and ameliorate ageism.

Furthermore, management support (i.e., being thanked for a job well-done; receiving support in the form of feedback on work performance) is the only work-related factor analyzed significantly associated with good care. Consistent with previous research, supporting capacities of supervisors towards their subordinates, plays a role of major importance [19]. Sufficient support for professionals and colleagues should be guaranteed in a friendly and reinforcing work atmosphere [18]. Practically, our results support the notion that researchers, chairs, supervisors, and nursing home professionals may need to focus on social support. Collective support (managerial and coworker social support) may provide the individual with more opportunities to perceive improved control, thereby improving good care.

Most nursing assistants take this employment because of not finding a job in their original occupation [32]. Workplace stress can be especially problematic for nursing aides or nursing assistants [32,60]. Contrary to our hypothesis, there were no differences on the good care ratings on the GCS-NH among positions in facilities. No association was found between good care and both being a nursing assistant or being a technician in the last step of the regression analysis. The correlation of good care with being a nursing assistant is no longer significant when work-related and personal resources are considered. These results may be explained by mediation effects. For example, being a technician may lead to receiving higher management support, and higher personal resources (i.e., more personal accomplishment and less depersonalization, and negative old age stereotypes). Previous studies have found less management support in nursing assistants [60]. Nursing assistants experienced higher levels of burnout and negative stereotypes [32,60] that may be related to less effective good care ability, involving less use of humanization, no infantilization, respect, and empowerment strategies.

This is one of the first studies to analyze the impact of personal and work-related resources on IOA good care. However, the different effects of management support, depersonalization, personal accomplishment, and negative old age stereotypes on good care for IOA should be studied further.

The results of the present study should be interpreted in light of its limitation involved in cross-sectional designs. We cannot make causal inferences because a cross-sectional study can only test associations between the variables. Further longitudinal research is needed to analyze this model of IOA good care. Moreover, intervention studies targeting work-related and personal resources might help to determine causality between work-related and personal resources and good care.

In addition to the cross-sectional design, this study has the following limitations. First, regarding the data-collection method through a self-reported survey, social desirability may have affected nursing staff´s answers by showing what the employer expects them to respond instead of their true feelings or impressions. Second, our findings cannot be generalized because of the use of a non-probability sample. A more representative sample of nursing home professionals should be included in future studies to provide a more complex view of good care, thereby advancing our knowledge. Third, data on residents and their relatives’ impressions were not collected. Future studies could be based on our results and go further by examining residents’ and their relatives’ concepts about nursing home professionals’ good care. Fourth, even though the regression model explained 31% of good care, this means that there are still additional factors influencing the IAO good staff that remain to be explored.

## 5. Conclusions

Despite these limitations, this study provides relevant information about the effect of personal and work-related variables on good care in front-line care nursing home professionals. In summary, in addition to management support, some personal issues—such as personal accomplishment, depersonalization, and negative old age stereotypes—seem to be relevant for explaining good care for IOA at nursing homes.

Good care seems to be related to work related factors and personal resources that may have to do with a negative view of aging, such as perceiving older adults as less capable, and with burnout feelings and management support. The data from this study suggests that it is not mainly work-related factors themselves but having positive perceptions of aging and less burnout perceptions that are related to good care for IOA. Nursing staff with positive perceptions of aging develop a better IOA care. Furthermore, nursing staff with engagement—characterized by energy, implication or commitment, and efficiency—may be regarded as the opposite to burnout, and seem to be more connected to IOA good care.

Policymakers and practitioners could consider the following aspects. First, promoting good care in nursing homes should begin by working on personal variables. The aim would be reducing staff burnout, a highly prevalent variable in nursing assistants working in long-term facilities for older people [32]. Professionals´ burnout should be sanctioned at the policy levels. Increasing the levels of personal accomplishment, and improving personalization attitudes may contribute to tackling burnout, which may, in turn, influence their good care to the residents. Second, modifying professionals´ attitudes towards older people can prevent negative old-age stereotypes. Burnout and negative stereotypes are modifiable through support, education, supervision, and other well-established means. Third, management support is a key point when changing the organization in the nursing home. It is also essential to assess the effect of these changes in IOA good care. Each nursing home should have clear policies to report and promote good care for its residents. Person-centered care and good care are wide constructs with relevant joint points. However, they are not exactly the same [61]. Good care is everyone’s business [44]. The theory of ‘doing good care’ involves anticipatory caring, and momentary caring [62]. Good care conveys to IOA that they are important. Individually, each good care behavior could have a positive impact. Collectively, they have the potential to result in a sense of strengths, optimism, and self-esteem [45]. Good care is both value reinforcing (it allows nursing home professionals to support the value of personhood) and an ethical expression (it is good to work with residents who are dependent and fragile). The essential test of this care is recognizing the uniqueness of the other. All of this, assuming that a holistic approach considers also factors associated with quality of life in IOA (control and autonomy, pleasure, and self-realization) [63].

The recent COVID-19 pandemic has shown the importance of having a well-organized health system, enough flexible to adapt to the people´s needs not only to respond to emerging threats, but also to cope with chronic diseases. COVID-19 outbreak had a limited impact on older adults’ psychological wellbeing [64]. However, IOA do not have usually an alternative home (the long-term center is their home), making the nursing homes an essential service and a priority for the whole of society. The preventive measurements of the lockdown of residences, sectorization of spaces, and isolation of residents has affected to good care during COVID-19 outbreak. The present study highlights the influence of nursing staff personal factors (a positive perception of aging and personal accomplishment and personalization) on good care. Nursing staff with lower levels of old age stereotypes and depersonalization will probably inform residents, and their relatives, with understandable language, answer all questions, and repeat information when necessary, about the emergency of exceptional situations and the existing COVID’s protocols (i.e., regarding referrals to health services, test for COVID-19, visit conditions). Furthermore, nursing staff with higher levels of personal accomplishment will probably discover new forms of working during COVID-19 outbreak (i.e., identify circuits in nursing homes that allow the development of routines basic mobility for IOA; guarantee the monitoring of educational and social work activities to the IOA). Nevertheless, more research is needed on this topic.

## Figures and Tables

**Table 1:** Sample characteristics (N = 208).

Variables	M (SD)/*n* (%)
Age, M (SD)	39.28 (11.85)
Gender (% male)	28 (13.5%)
Marital status (% married)	89 (42.8%)
Education (% Lower than high school diploma)	41 (19.7%)
Position in facility	
Nursing assistants (IOA care providers)	149 (71.6%)
Interdisciplinary team technicians	59 (28.4%)
Nursing home equipment and facilities (% adequate)	151 (72.6%)
Non-consistent assignment of staff (turnover) (% yes)	166 (79.8%)
Staff-to-resident ratio (% adequate)	51 (24.5%)
Good care, M (SD)	58 (9.30)
Professional quality of life, M (SD)	
Management support	6.05 (1.71)
Work overload	4.84 (1.96)
Intrinsic motivation	8.63 (0.98)
Burnout, M (SD)	
Emotional exhaustion	10.78 (10.35)
Depersonalization	5.03 (4.83)
Personal accomplishment	40.52 (7.52)
Negative old age stereotypes, M (SD)	33.23 (8.10)

Data are presented as mean (SD), or *n* (%). Professional quality of life = Professional quality of life, PQL-35 Questionnaire; Burnout = Maslach Burnout Inventory; Negative old age stereotypes = Negative Stereotypes Towards Ageing Questionnaire; Good Care = Good Care Scale in Nursing Homes.

**Table 2:** Hierarchical regression analysis examining the associations between assessed variables and good care

Variables	Step 1			Step 2			Step 3		
	B	SE	B	B	SE	B	B	SE	B
Position in facility (0 = Nursing assistants)	7.111	1.931	0.256 ***	3.447	1.929	0.124	0.932	1.975	0.034
Staff-to-resident ratio (1= adequate)				0.957	1.975	0.034	0.773	2.235	0.027
Turnover) (1= yes)				0.783	2.174	0.024	0.997	2.080	0.031
Nursing home equipment and facilities (1 = adequate)				1.607	2.033	.059	0.696	1.929	0.025
Management support				0.208	0.043	0.372 ***	0.145	0.052	0.259 **
Work overload							0.005	0.054	0.008
Intrinsic motivation							−0.042	0.110	−0.030
Emotional exhaustion							0.016	0.117	0.013
Depersonalization							−0.478	0.215	−0.186 *
Personal accomplishment							0.400	0.114	0.243 **
Negative old age stereotypes, M (SD)							−0.370	0.101	−0.242 ***
Change in adjusted R^2^	0.061 ***			0.139 ***			0.111 ***		

* *p* < 0.05. ** *p* < 0.01. *** *p* < 0.001.

## Data Availability

Upon reasonable request, data used for this publication can be re-analyzed under the terms and conditions of the Spanish data protection laws. The datasets used and/or analyzed during the current study are available from the senior author at jlopezm@ceu.es.
